# Biexciton and Singlet Trion Upconvert Exciton Photoluminescence in a MoSe_2_ Monolayer Supported by Acoustic and Optical K-Valley Phonons

**DOI:** 10.1021/acs.jpclett.3c01982

**Published:** 2023-09-21

**Authors:** Joanna Jadczak, Joerg Debus, Justyna Olejnik, Ching-Hwa Ho, Kenji Watanabe, Takashi Taniguchi, Leszek Bryja

**Affiliations:** † Department of Experimental Physics, Wrocław University of Science and Technology, Wybrzeże Wyspiańskiego 27, 50-370 Wrocław, Poland; ‡ Department of Physics, TU Dortmund University, 44227 Dortmund, Germany; ¶ Graduate Institute of Applied Science and Technology, National Taiwan University of Science and Technology, Taipei 106, Taiwan; § National Institute for Materials Science, Tsukuba, Ibaraki 305-0044, Japan

## Abstract

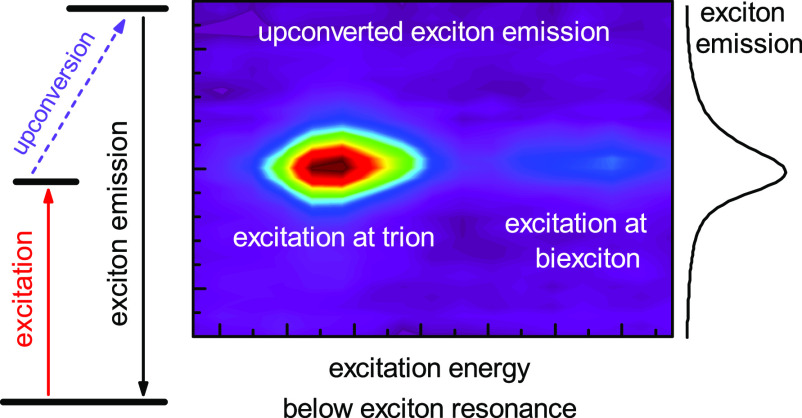

Transition metal dichalcogenide monolayers represent unique platforms for studying both electronic and phononic interactions as well as intra- and intervalley exciton complexes. Here, we investigate the upconversion of exciton photoluminescence in MoSe_2_ monolayers. Within the nominal transparency window of MoSe_2_ the exciton emission is enhanced for resonantly addressing the spin-singlet negative trion and neutral biexciton at a few tens of meV below the neutral exciton transition. We identify that the A′_1_ optical phonon at the K valley provides the energy gain in the upconversion process at the trion resonance, while ZA(K) phonons with their spin- and valley-switching properties support the biexciton driven upconversion of the exciton emission. Interestingly, the latter upconversion process yields unpolarized exciton photoluminescence, while the former also leads to circularly polarized emission. Our study highlights high-order exciton complexes interacting with optical and acoustic K-valley phonons and upconverting light into the bright exciton.

Transition metal dichalcogenide (TMDC) monolayers are direct band gap semiconductors with unique optical and spin-valley properties.^1^ Their two-dimensional (2D) nature and the reduced dielectric screening of the Coulomb interaction allow for the formation of excitons with a binding energy of hundreds of meV^2,3^ and high-order excitonic complexes, such as trions and biexcitons with binding energies of tens of meV.^4−12^ Moreover, due to the strong spin–orbit coupling in TMDC monolayers and the resulting valley-contrasting spin splitting at the K valleys, the excitonic complexes possess both the spin as well as valley degree of freedom.^1^

In optically darkish systems, such as WS_2_ and WSe_2_ monolayers, the electric-dipole forbidden (optically dark) exciton state is positioned at lower energy than the optically active exciton state. On the contrary, in MoSe_2_ monolayers the bright exciton state is at the lowest energy. These level hierarchies as well as exciton–phonon interactions were studied due to the progress in elaborating high-quality hBN-encapsulated TMDC monolayers.^13^ In this context, upconversion photoluminescence (PL) spectroscopy recently provided thorough insights into exciton–exciton and exciton–phonon interactions and was successfully employed to reveal the coupling between various excitonic complexes in the optically darkish hBN-encapsulated WSe_2_ and WS_2_ monolayers. In particular, upconversion of light from dark excitons, trions, and biexcitons to bright excitons was observed.^14−16^

In the n-type MoSe_2_ monolayer, the lowest energy trion configuration is the negative intervalley spin-singlet state (T_S_). Interestingly, the T_S_ state typically dominates the PL spectra in MoSe_2_ monolayers at low temperatures but is dramatically weakened with increasing temperature. In contrast to that, in Mo(S_*y*_Se_1–*y*_)_2_ alloys with a sulfur mole content up to *y* = 0.5, the trion emission is also robust at elevated temperatures.^8^ This observation was attributed to a strong increase in the exciton–trion coupling strength and to a rising 2D electron gas concentration caused by an increasing sulfur content.^8^ The enhanced exciton–trion coupling was realized by tuning phonon energies to the trion binding energy in the Mo(S_*y*_Se_1–*y*_)_2_ alloys. Also, the coherent and incoherent nature of the exciton–trion coupling and relevant time scales in MoSe_2_ monolayers were revealed by optical 2D coherent spectroscopy; it demonstrated an efficient energy transfer via phonon-assisted exciton-to-trion downconversion within 2–3 ps and trion-to-exciton upconversion in 8 ps.^17^ The exciton–trion interaction in TMDC monolayers may be alternatively probed in upconversion (UPC) PL experiments.^14,18^ The excess energy required for the UPC process may be taken from phonons or resident electrons in the monolayer.^15^ Hence, the UPC PL provides information on both the energy spectra of the TMDCs as well as the scattering mechanism related to exciton–exciton, exciton–electron, and exciton–phonon interactions.

Here, we probe the upconversion photoluminescence in an optically bright hBN-encapsulated MoSe_2_ monolayer system. UPC PL excitation reveals two pronounced resonances below the neutral 1s A-exciton (X). The resonance detected at an energy of about 25 meV below X coincides with the PL peak and binding energy of the singlet trion T_S_. The second resonance at −18 meV with respect to the exciton transition is attributed to the neutral biexciton (XX^0^), which was previously identified in a MoSe_2_ monolayer using polarization-resolved 2D coherent spectroscopy.^10^ We propose that the energy gains required in the UPC of the exciton PL originate from different interactions, including optical or acoustic phonons at the K valley. The upconversion of light from T_S_ and XX^0^ into X is observed only for samples with a relatively weak electron concentration and is enhanced at elevated temperatures. We also evaluate the UPC as a function of the incident laser power and demonstrate specific polarization characteristics of the upconverted exciton emission. In contrast to tungsten-based structures, probing of upconverted exciton PL in MoSe_2_ requires a low number of resident electrons and is realized only in hBN-encapsulated samples. Moreover, as biexcitons are challenging to be identified unambiguously using linear optical spectroscopy methods and were detected only in WS_2_ and WSe_2_ monolayers so far in regular PL experiments, upconversion is an alternative route to address, for example, biexcitons in TMDC monolayers, particularly in MoSe_2_. UPC further provides information on the energies of bound carrier complexes in TMDCs and scattering processes related to exciton–electron and exciton–phonon interactions.

In Figure 1a, intensity normalized PL spectra are shown for an uncapped MoSe_2_ monolayer placed on an hBN substrate (MoSe_2_/hBN) and for an hBN-encapsulated MoSe_2_ monolayer (hBN/MoSe_2_/hBN). The spectra were measured at 7 K and were excited nonresonantly by laser light having an energy of 2.33 eV. Since the energy *E*_X_ of the exciton PL peak changes from flake to flake between 1.627 and 1.638 eV, the energy scale of each spectrum is referred to the exciton energy; accordingly, the energy difference *E* – *E*_X_ is chosen for the horizontal axis. The predominant peak which is about 26 or 30 meV below the exciton PL peak is attributed to the spin-singlet negative trion T_S_ whose binding energy lies in this energy range.^5^ While the neutral exciton recombination in MoSe_2_ yields a symmetric peak, the trion PL is asymmetric with a broad low-energy flank which is attributed to an electron recoil effect for trions.^19^ The doping level of the samples could be estimated by the intensities and relative energy positions *ΔE*_X–T_ of the exciton and trion PL peaks. On the one hand, the X emission intensity in the MoSe_2_/hBN structure is significantly lower than that in the hBN/MoSe_2_/hBN sample and, on the other hand, *ΔE*_X–T_ amounts to 30 and 26 meV, respectively. These differences indicate a higher electron density in the uncapped structure. On the basis of the exciton–trion energy splittings *ΔE*_X–T_ and gate-dependent optical characteristics of MoSe_2_,^20^ we estimate the electron density to be about 2 × 10^10^ cm^–2^ in the encapsulated monolayer, whereas in the uncapped monolayer it is in the order of 10^12^ cm^–2^. Additionally, in Figure 1b we compare the reflectance contrast (RC) spectra of both samples excited by white light. As clearly seen, only for MoSe_2_/hBN a weak negative trion resonance is resolved, which underlines the presence of a significant electron doping.^20^ For hBN/MoSe_2_/hBN, the exciton resonance is sharp and intense due to the low electron doping level and the encapsulation, which protects the monolayer from charge transfers and local electric field fluctuations.

**Figure 1:**
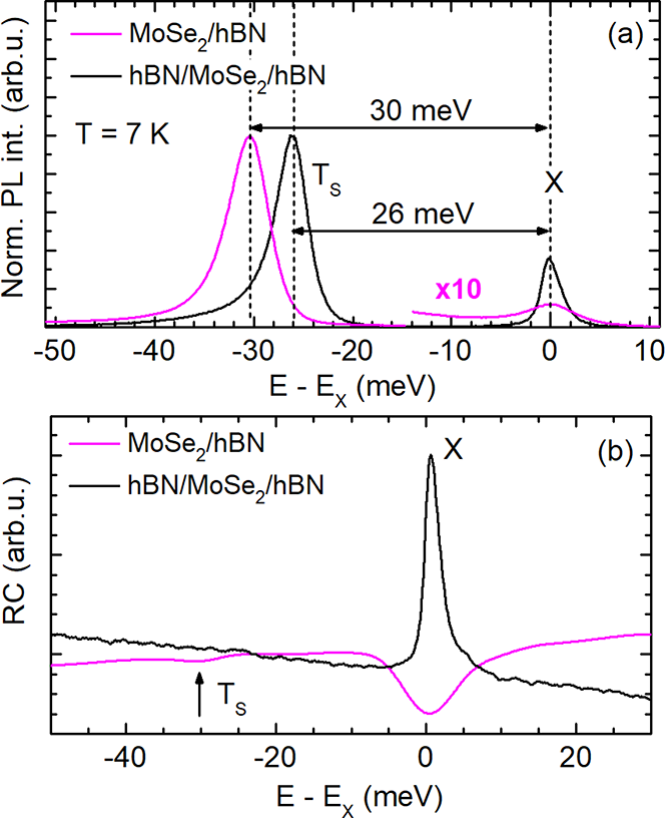
(a) PL spectra of MoSe_2_/hBN and hBN/MoSe_2_/hBN van der Waals heterostructures measured at *T* = 7 K. The energy splitting between the exciton and singlet trion PL lines is marked by a horizontal arrow. (b) RC spectra for both structures measured at 7 K.

We now focus on the upconversion of the neutral exciton PL in the MoSe_2_ monolayer, for excitation energies below the neutral exciton in the nominal MoSe_2_ transparency range, and demonstrate its dependence on variations of the temperature, laser power, and polarization. In the following, we present the data for the hBN/MoSe_2_/hBN heterostructure. For the MoSe_2_/hBN structure, we observed only negligibly weak upconverted X PL.

The regular and upconverted PL of the hBN/MoSe_2_/hBN structure recorded at 7 K is demonstrated in Figure 2. In Figure 2a, the PL spectrum and the energy range that is resonantly excited for the UPC PL are shown. The latter marked by vertical arrows goes from about −15 meV to the low-energy flank of the trion PL at about −29 meV. For a UPC photoluminescence excitation (PLE) spectrum, the neutral exciton PL is monitored during the variation of the laser excitation energy *E*_exc_. The color map in Figure 2b displays the UPC PLE spectra as a function of the excitation energy detuned from *E*_X_, while the integrated exciton UPC PL *I*_X,UPC_ is shown in Figure 2c. The UPC energy gain is given by the energy difference |*E*_exc_ – *E*_X_|. Both the color map and the integrated exciton UPC PL clearly exhibit a prominent resonance at an energy gain of about 25 meV. This corresponds to the binding energy of the spin-singlet negative trion T_S_. A second weak resonance is observed at an energy gain of about 18 meV. This enhancement of the exciton PL may originate from the UPC process involving the neutral biexciton XX^0^ whose binding energy lies in this range.^10^

**Figure 2:**
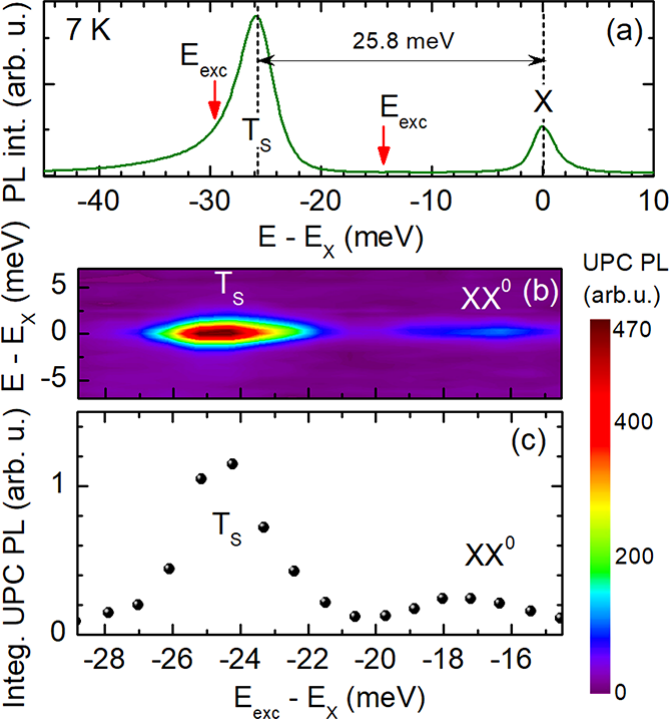
(a) PL spectrum of the hBN/MoSe_2_/hBN structure detected at 7 K, for nonresonant laser excitation at 2.33 eV. (b) Color map of the UPC PLE spectra with a detection range of ±6 meV at exciton resonance *E*_X_; *T* = 7 K. (c) Integrated UPC PL of the neutral exciton for excitation energies ranging from −29 to about −15 meV with respect to the X resonance.

In order to gain further insight into the exciton emission upconverted from the trion T_S_ and biexciton XX^0^ we perform comparative temperature-dependent PL and UPC PLE measurements. In Figures 3a–f the PL spectra, the UPC PLE spectra, and the integrated UPC PL measured at 40 and 80 K are presented. At these temperatures, we tune the excitation energy through the biexciton and singlet trion resonances of the MoSe_2_ monolayer, as marked by the red arrows in the PL spectra of panels a and b, and detect the response of the X emission. The resulting UPC PLE spectra are shown in panels c and d, and the integrated UPC PL *I*_X,UPC_ of the X is presented in panels e and f. Additionally, Figure 3g provides an overview about the integrated intensities of the X and T_S_ PL peaks (black symbols) and the integrated X PL upconverted from the XX^0^ and T_S_ states (red symbols) as a function of temperature from 7 to 80 K. Up to 50 K, the PL intensity of the negative trion exceeds that of the exciton, while both lines become weaker with increasing temperature. By comparison, the upconverted X PL is enhanced with rising temperature: *I*_X,UPC_ is maximum at about 50 K, for resonantly exciting T_S_, and is reduced by further increasing the temperature. For exciting XX^0^, *I*_X,UPC_ exhibits a maximum at about 60 K. This thermal behavior differs from that observed in WS_2_ monolayers,^16,18^ where the upconverted PL of the exciton becomes significantly intensified with rising temperature and even exceeds the regular PL intensity.

**Figure 3:**
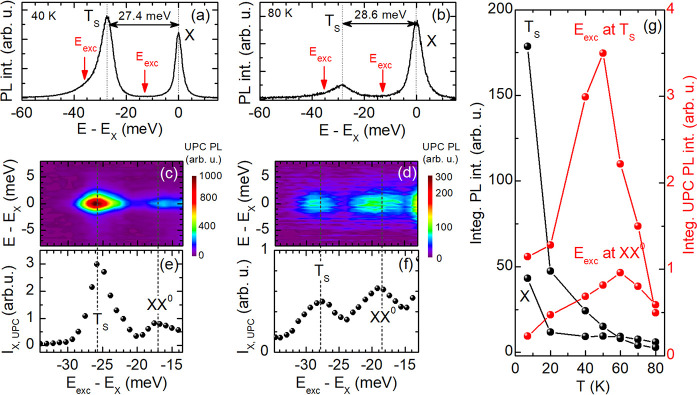
PL spectra of the hBN-encapsulated MoSe_2_ monolayer recorded at (a) 40 and (b) 80 K. Color maps of the UPC PLE spectra for varying energy gain measured at (c) 40 and (d) 80 K. (e and f) Integrated UPC PL of X as a function of the energy gain given at 40 and 80 K, respectively. (g) Temperature dependence of (left scale) integrated PL intensities and (right scale) *I*_X,UPC_ excited at T_S_ and XX^0^.

We further study the dependence of the upconverted X PL on the incident laser power *P*. It will provide insight into the character of the UPC resonances, in addition to the prior assignment of the resonances according to the binding energies of the negative trion and neutral biexciton. Typical UPC PLE spectra recorded for 10, 6, and 4 mW are depicted in Figure 4a,b,c, respectively. The evolution of the integrated UPC exciton PL as a function of *P*, for resonantly addressing the negative trion and biexciton, is given in panel d using a double-logarithmic presentation. *I*_X,UPC_ depends nonlinearly on the laser power which is evaluated from the slopes α of 2.08 and 1.66, for exciting the XX^0^ and T_S_ resonances, respectively. The practically quadratic power dependence (α_XX^0^_ = 2.08) of *I*_X,UPC_, for *E*_exc_ – *E*_X_ = −18 meV, characterizes a process in which a biexciton is involved. The power-dependent evolution of the X PL intensity upconverted from T_S_ is described by α_T_S__ = 1.66, which reflects the power-dependent slope of the trion regular PL intensity.^21^

**Figure 4:**
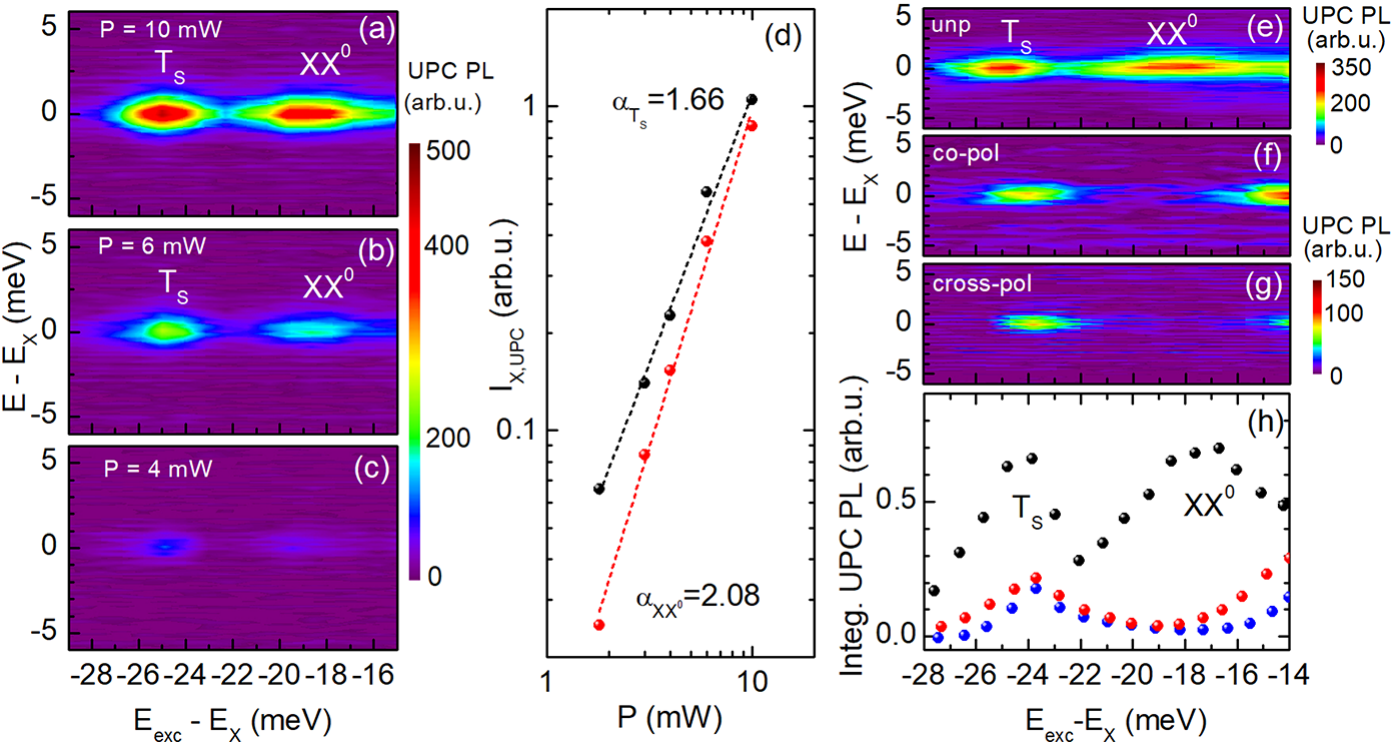
UPC PLE spectra as color maps obtained at (a) 10 mW, (b) 6 mW, and (c) 4 mW laser power. (d) The integrated UPC PL of the exciton is shown as a function of the laser power, for addressing the XX^0^ (red circles) and T_S_ (black circles). (e–g) Unpolarized and circular-polarization resolved UPC PLE spectra measured at 7 K and *P* = 8 mW. The color scale at the top right belongs to panel e; the bottom-right scale refers to panels f and g. (h) Integrated UPC PL of X as a function of the energy gain, for the different polarization configurations. The black colored data points represent the unpolarized case, while the red (blue) symbols display the copolarized (cross-polarized) configuration.

Finally, we elucidate the polarization properties of the exciton PL upconverted from the negative trion and neutral biexciton states, yielding additional details on the UPC mechanisms, which are discussed in the following section. In Figure 4e the UPC PLE spectra are demonstrated for linearly polarized excitation and unpolarized detection, while in Figure 4f,g the incident light and the emission are cocircularly and cross-circularly polarized, respectively. Figure 4h displays *I*_X,UPC_ as a function of the polarization configurations for excitation energies ranging from −28 to −14 meV in proximity to the X. The upconverted X PL is predominantly unpolarized, for tuning the excitation energy to the biexciton resonance, while in the circularly polarized spectra *I*_X,UPC_ is negligibly weak. By comparison, the exciton PL upconverted from the negative trion T_S_ is slightly polarized (+10%) with regard to the laser light polarization, while its intensity is also high in the unpolarized configuration. It is worthwhile to mention that *I*_X,UPC_ becomes significantly polarized (+50%) for reducing the energy gain to 14 meV. It is consistent with results obtained for the regular exciton PL whose circular polarization is rather low compared to other TMDCs^22^ and is enhanced for approaching the excitation energy of the A-exciton resonance.^23,24^

The experimental observations performed in the nominal transparency window of MoSe_2_ are summarized in the following: (a) only the hBN-encapsulated MoSe_2_ monolayer exhibits pronounced UPC PL of the neutral exciton (1s A-exciton); (b) the X PL is enhanced for resonantly addressing the spin-singlet negative trion at an energy gain of about 25 meV and the neutral biexciton at about 18 meV; (c) the UPC PL possesses intensity maxima at elevated temperatures; (d) the UPC PL intensity is scaled nonlinearly with the incident laser power; and (e) the UPC PL of the exciton is clearly unpolarized at the XX^0^ resonance and exhibits a slightly positive circular polarization at the T_S_ resonance.

In contrast to WSe_2_ and WS_2_ monolayers in which the lowest exciton state is optically forbidden (spin-dark)^25^ at the K valleys, MoSe_2_ monolayers are characterized by spin-allowed neutral excitons as energetically lowest states at the K_+_ and K_–_ valleys. The bright exciton at the K_+_ (K_–_) valley is excited by σ^+^ (σ^–^) circularly polarized light in accordance with a valley index of either +1 or −1. Here, the upconversion of the exciton emission is initiated by optically creating either the neutral biexciton XX^0^ or the negative trion T_S_. The difference between the neutral exciton energy and the incident laser light energy corresponds to the binding energy of the excitonic complex involved in the UPC process. An individual number of interactions is followed by the final exciton annihilation. The overall efficiency of the UPC is governed by the order of the process, whereby spin- and momentum-conserving scattering or exchange interaction processes are favored.

We first consider the exciton PL UPC involving the neutral biexciton which is observed at an optical excitation energy of about *ΔE*_XX^0^_ = 18 meV below the X transition (corresponding to the XX^0^ binding energy) and in the presence of only a low number of resident electrons (close to the neutrality point). Moreover, the energy difference (energy gain) lies within the range of phonon energies, and the UPC is particularly pronounced at elevated temperatures. These aspects indicate the significance of phonon contributions without a disturbing impact of additional electrons (due to, e.g., exchange interaction). Additionally, the quadratic power dependence is also characteristic for neutral biexcitons. Besides the specific temperature dependence and low energy gain, the strong nonlinear power dependence rules out a potential two-photon absorption with a real biexciton state and an Auger-like process, as demonstrated by ref (26). As shown in Figure 5a, we thus propose that initially (1) a neutral biexciton with spin-up electron and hole at the K_+_ and spin-down electron and hole at the K_–_ valley are excited, whereby the net angular momentum transfer is zero, in agreement with the linearly polarized incident light. Afterward, (2) each electron interacts with the zone-corner flexural acoustic ZA(K) phonon mode of about *E*_ZA_ = 17.5 meV (4.2 THz) energy.^27^ Hence, each electron is scattered to the opposite valley (*K*_+_ → *K*_–_, *K*_+_ → *K*_–_),^28^ under reversal of its spin so that in total the spin angular momentum is not changed and the crystal momentum is conserved. In accordance with the Elliot–Yafet mechanism the phonon in the spin-flip process must possess an odd parity under mirror-symmetry operation.^29^ In contrast to the longitudinal and transversal acoustic phonon modes, the ZA(K) phonon fulfills this criterion.^27^ The phonon absorption provides the energy 2*E*_ZA_ to the carrier complex which is about twice the binding energy (2*ΔE*_XX^0^_). Therefore, we propose that after the electron–phonon interaction, the electrons relax to the minimum of their conduction sub-bands, as sketched in the right scheme of Figure 5a. The electron–phonon interaction with the subsequent electron relaxation adds dispersion to the resonance energy so that from about *E*_exc_ – *E*_X_ = −20 meV until −16 meV the XX^0^ resonance is observed. Finally, (3) both excitons recombine, giving rise to emission which is unpolarized. Since the upconverted X PL is not observed in any circular polarization setting of the detection path, for linearly polarized excitation, we conclude that the final state composed of two bright excitons residing at the K_+_ and K_–_ valleys is a coherent intervalley (superposition) state.

**Figure 5:**
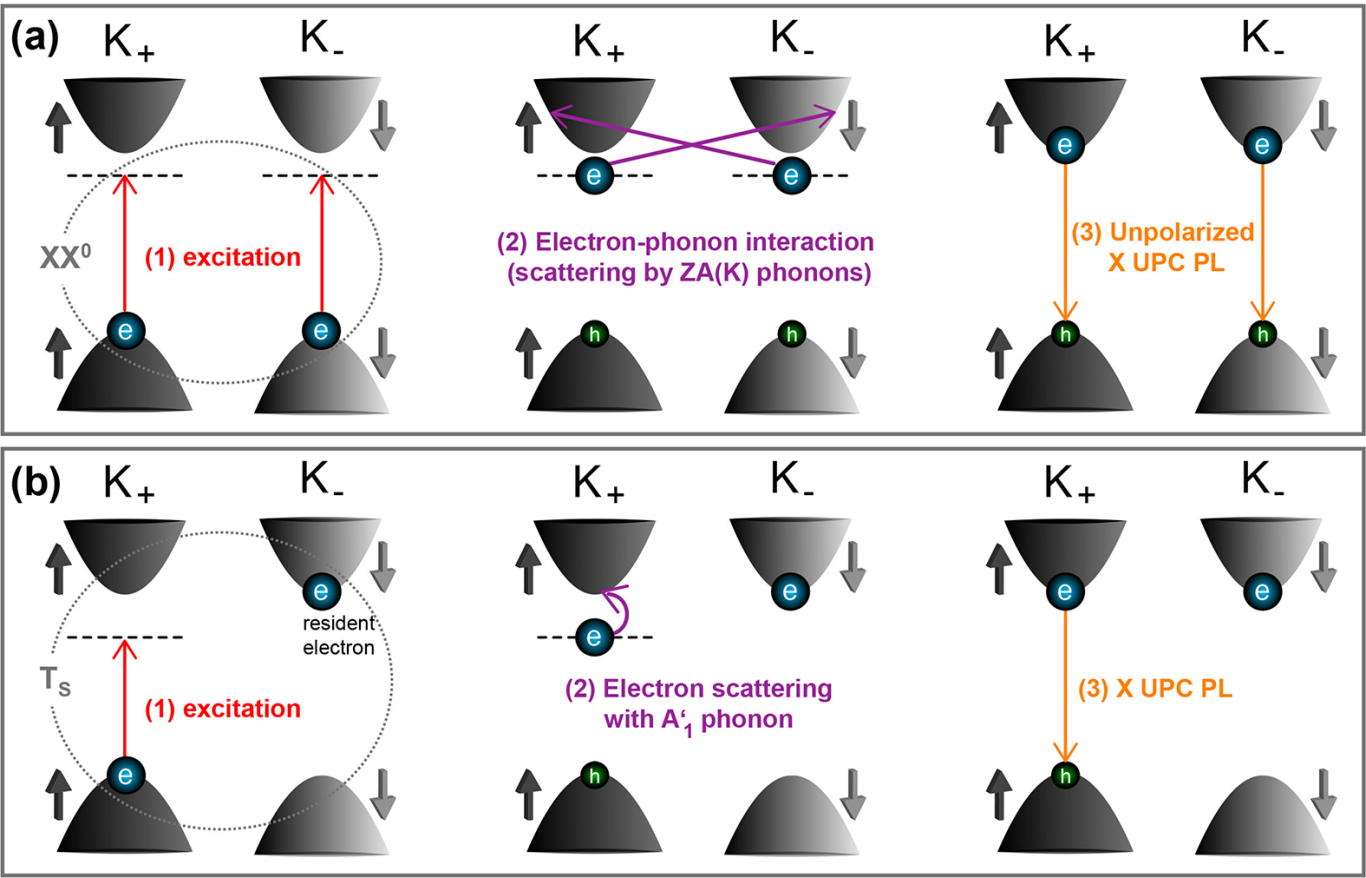
Schematic presentation of the mechanisms for upconverting the exciton PL by the (a) neutral biexciton and (b) spin-singlet trion. Each UPC process is sequentially sketched by going from the left to the right side. The single-particle picture is chosen where an electron (a hole) is illustrated by a blue (green) sphere. Only the energetically lowest conduction subband and highest valence subband are shown.

We take into account the neutral biexciton in MoSe_2_ composed of two spin-allowed intravalley excitons residing at the K_+_ and K_–_ valley, respectively. This intervalley XX^0^ configuration is, due to Pauli blocking, energetically favored against an intravalley configuration in which a bright and dark exciton are both excited in the same valley.^11^

For the exciton emission upconverted from the spin-singlet trion, we propose the following spin-conserving process. As shown in Figure 5b, for σ^+^ polarized excitation and detection, (1) the T_S_ state is excited with a spin-up electron and hole at the K_+_ valley as well as a spin-down resident electron at the K_–_ valley. (2) As only for low electron concentration the X PL is upconverted from T_S_, we propose that the spin-up photoelectron is scattered by the A′_1_ phonon to the energetically lowest subband at the K_+_ valley. The A′_1_ optical phonon at the K valleys has an energy of about 25 meV (6 THz),^27^ which well fits to the T_S_ binding energy. While the resident electron remains at the spin-down subband of the K^–^ valley, the spin-up photoelectron as well as the hole at the K^+^ valley recombine, resulting in σ^+^ polarized exciton emission. The X PL upconverted from the negative trion is both copolarized as well as slightly cross-polarized. The cross-polarization may hint at a stimulated exciton (boson) scattering.^26^ Alternatively, due to intervalley scattering of the hole including a spin reversal,^23^ the recombination of the electron and hole at the K_–_ valley leads to σ^–^ polarized exciton emission which also explains the emission in the cross-polarized configuration.

The contribution of phonon modes to UPC processes in TMDCs strongly depends on temperature.^18,30^ Recent theoretical calculations have shown that in MoSe_2_ the upconversion rate is significantly higher than that in WSe_2_.^30^ It results in a stronger electron–phonon interaction and a shorter thermal average upconversion time, which is about eight times faster in MoSe_2_ than in WSe_2_.^30^ Moreover, in a MoSe_2_ monolayer the thermal average upconversion time decreases strongly with increasing temperature; at 60 K it is about 10 times smaller than at 7 K.^30^ This thermal behavior supports the explanation of the temperature dependences observed in our experiments and the UPC mechanisms, including optical and zone-edge acoustic phonons. The temperature-dependent growth of the exciton UPC PL is likely governed by an increased phonon population, leading to a more probable phonon-mediated scattering of the electrons. By comparison, the thermally induced PL decrease in the MoSe_2_ monolayer may be caused by an intravalley scattering with acoustic phonons.^31,32^

For an hBN-encapsulated MoSe_2_ monolayer with a relatively low concentration of resident electrons (2 × 10^10^ cm^–2^), we have demonstrated the upconversion of the 1s A-exciton PL by the neutral biexciton and singlet trion, respectively. In the nominal transparency window of MoSe_2_ the X PL is observed for resonantly addressing T_S_ (XX^0^) at an energy of about 25 meV (18 meV) below the neutral exciton resonance. The upconverted X PL is enhanced nonlinearly with the incident laser power and also at elevated temperatures of 50–60 K. The latter is attributed to an increased phonon population giving rise to a high phonon-mediated scattering rate of the electrons. Additionally, the UPC PL of the exciton is unpolarized at the XX^0^ resonance and displays a slight circular polarization at the T_S_ resonance. The mechanism of the exciton PL upconverted by the neutral biexciton is attributed to the interaction of the photocreated electrons at the K_+_ and K_–_ valleys with zone-corner flexural acoustic ZA(K) phonons. They scatter the electrons to opposite valleys under reversal of their spins. Finally, two bright excitons residing at the K_+_ and K_–_ valleys recombine leading to unpolarized emission at the neutral exciton energy. The UPC of the X PL via the spin-singlet negative trion is assigned to a spin- and valley-conserving scattering process of the photocreated electron with the optical A′_1_ phonon mode whose energy at the K valleys matches the energy difference between the singlet trion and the neutral exciton.

Our results extend the current discussion about interactions of electrons with both optical and acoustic phonons at the K valleys and their role in the upconversion of exciton emission in MoSe_2_ monolayers. We also provide further insights into resonant exciton–trion and exciton–biexciton couplings for optically exciting a 2D material within its nominal transparency range.

## Materials and Methods

MoSe_2_ crystals were grown by a chemical vapor transport technique. Prior to the crystal growth, the powdered compounds were prepared from the elements Mo (purity: 99.99%) and Se (99.999%) by reaction at 1000 °C for 10 days in quartz ampules. The mixture was slowly heated to 1000 °C. The chemical transport was achieved with I_2_ as transport agent having a concentration of about 5 mg/cm^3^. The growth temperature was gradually changed from 1030° to 980 °C, with a temperature gradient of 3 °C/cm and a growth time of 20 days. The crystals had the shape of thin-layered plates with thicknesses and surface areas ranging from 20 to 1000 μm and from 20 to 100 mm^2^, respectively.

We prepared van der Waals hBN-encapsulated MoSe_2_ heterostructures using high-purity hexagonal boron nitride (hBN) and Si substrates (300 nm SiO_2_). The monolayers were mechanically exfoliated from the MoSe_2_ bulk crystals using the deterministic all-dry stamping method, similar to Castellanos-Gomez et al.^33^ A MoSe_2_ monolayer and hBN crystals were first exfoliated on a flexible PDMS gel-film stamp rigidly attached to a glass slide. The thicknesses of the hBN flakes were about 200 nm for the bottom layer in MoSe_2_/hBN structures and about 100 nm for the bottom layer and about 2 nm for the top layer, respectively, in hBN-encapsulated structures. During the transfer process, the substrate and the stamp were placed below an optical microscope equipped with an XYZ positioning stage. A long-working distance microscope objective enabled us to locate and deterministically transfer selected flakes to the substrate. After each transfer step the sample was heated to about 180 °C for 20 min in air. After the last layer was added to the sample, a final thermal annealing was performed in air for 2 h at about 200 °C.

For the PL and UPC PLE experiments, the samples were mounted on the coldfinger of a nonvibrating closed-cycle helium cryostat, in which the temperature could be varied from 7 to 350 K. The PL was excited by the second harmonic 532 nm (2.33 eV) of a continuous-wave single-mode Nd:YAG laser. The UPC PLE was excited by a continuous-wave Ti:sapphire laser whose emission was tunable in the range from 760 to 780 nm. The laser beam was focused on the sample under normal incidence using a high-resolution, long-working distance (WD = 10 mm, NA = 0.65) 50× microscope objective. The diameter of the excitation spot was about 1 μm. The emission from the sample was collected by the same microscope objective and was analyzed with a 0.5-m-focal length spectrometer equipped with a 600 lines/mm grating and a Peltier-cooled charged-coupled-device Si camera. The RC spectrum was measured at the same setup using a filament lamp as a light source. To eliminate the scattered laser light, a set of short- and long-pass edged filters was used. For the polarization-resolved experiments, a Glan-Thompson prism combined with a quarter-wave retardation plate was introduced in the excitation and detection path.
